# Early life environmental and pharmacological stressors result in persistent dysregulations of the serotonergic system

**DOI:** 10.3389/fnbeh.2015.00094

**Published:** 2015-04-27

**Authors:** Peiyan Wong, Ying Sze, Laura Jane Gray, Cecilia Chin Roei Chang, Shiwei Cai, Xiaodong Zhang

**Affiliations:** ^1^Neuroscience and Behavioral Disorders Program, Duke-NUS Graduate Medical School SingaporeSingapore, Singapore; ^2^Department of Pharmacology, Neuroscience Phenotyping Core, National University of SingaporeSingapore, Singapore; ^3^Department of Psychiatry and Behavioral Sciences, Duke University Medical CenterDurham, NC, USA; ^4^Department of Physiology, National University of SingaporeSingapore, Singapore

**Keywords:** TPH2, maternal separation, chronic mild stress, depression, anxiety, monoamine oxidase A, dexamethasone, serotonin

## Abstract

Dysregulations in the brain serotonergic system and exposure to environmental stressors have been implicated in the development of major depressive disorder. Here, we investigate the interactions between the stress and serotonergic systems by characterizing the behavioral and biochemical effects of chronic stress applied during early-life or adulthood in wild type (WT) mice and mice with deficient tryptophan hydroxylase 2 (TPH2) function. We showed that chronic mild stress applied in adulthood did not affect the behaviors and serotonin levels of WT and TPH2 knock-in (KI) mice. Whereas, maternal separation (MS) stress increased anxiety- and depressive-like behaviors of WT mice, with no detectable behavioral changes in TPH2 KI mice. Biochemically, we found that MS WT mice had reduced brain serotonin levels, which was attributed to increased expression of monoamine oxidase A (MAO A). The increased MAO A expression was detected in MS WT mice at 4 weeks old and adulthood. No change in TPH2 expression was detected. To determine whether a pharmacological stressor, dexamethasone (Dex), will result in similar biochemical results obtained from MS, we used an *in vitro* system, SH-SY5Y cells, and found that Dex treatment resulted in increased MAO A expression levels. We then treated WT mice with Dex for 5 days, either during postnatal days 7–11 or adulthood. Both groups of Dex treated WT mice had reduced basal corticosterone and glucocorticoid receptors expression levels. However, only Dex treatment during PND7–11 resulted in reduced serotonin levels and increased MAO A expression. Just as with MS WT mice, TPH2 expression in PND7–11 Dex-treated WT mice was unaffected. Taken together, our findings suggest that both environmental and pharmacological stressors affect the expression of MAO A, and not TPH2, when applied during the critical postnatal period. This leads to long-lasting perturbations in the serotonergic system, and results in anxiety- and depressive-like behaviors.

## Introduction

Increasing evidence suggest that stress is an important factor in the etiology of depression in humans (Hammen, 2005; Clark et al., 2007; Cohen et al., 2007; Heim and Binder, 2012). The normal physiological response to stress is the activation of the hypothalamic-pituitary-adrenal (HPA) axis. This results in the production of corticosteroids that bind to and activate mineralocorticoid and glucocorticoid receptors, which give rise to physiological and homeostatic changes downstream (De Kloet, 2004; Smith and Vale, 2006). However, constant activation of the stress response may cause dysregulations in the HPA axis, resulting in permanently altered circulating corticosterone levels (Lanfumey et al., 2008; Vreeburg et al., 2009; Frodl and O’Keane, 2013) or epigenetic changes in the expression of glucocorticoid receptors (Chung et al., 1999; Meaney and Szyf, 2005). In addition, there has been increasing consensus that the extent of adverse outcome following a stressful experience is largely dependent on the timing, duration and type of stressor (Avital and Richter-Levin, 2005; Tsoory et al., 2007; Lupien et al., 2009). In particular, early life stress seems to have long-lasting impacts on adult behavior. This has been attributed to the fact that the brain and HPA axis of developing animals are more vulnerable to stress-induced pre-programming (Matthews, 2002; Champagne et al., 2009; Huang, 2011; Banerjee et al., 2012).

Dysregulation of the serotonergic system, such as decreased serotonin synthesis caused by a rare mutation that compromises the functionality of the tryptophan hydroxylase (TPH) 2 gene (Zhang et al., 2004, 2005), has also been directly implicated in the pathophysiology of depression (Asberg et al., 1976; Gao et al., 2012; Jacobsen et al., 2012a). Interestingly, there are several interactions between the HPA axis and the serotonergic system (Fuller and Snoddy, 1980; Chaouloff, 1993; Dinan, 1996; McAllister-Williams et al., 1998; Porter et al., 2004; Heisler et al., 2007). For example, serotonergic cell bodies in the brain have increased glucocorticoid receptor expression (Lanfumey et al., 2008). In addition, direct manipulation of serotonin levels in the brain has also been shown to affect the expression of glucocorticoid receptors (Sémont et al., 2000). Thus, the serotonin signaling system appears to be a critical point of intersection between an environmentally driven stress response and genetic predisposition to depression (Caspi et al., 2003; Porter et al., 2004; Firk and Markus, 2007; Lanfumey et al., 2008; Alexander et al., 2009). In mice that had undergone chronic stress, those with deficient serotonin showed increased susceptibility to mood disorders as compared to wild type (WT) mice (Sachs et al., 2013, 2014; Gutknecht et al., 2015). In rhesus monkeys, a polymorphism in the tryptophan hydroxylase 2 (TPH2) gene, combined with early life experiences caused changes in the baseline cortisol levels and overactivation of the HPA axis, as well as behaviors such as aggressive displays (Chen et al., 2010). As such, stress and serotonergic abnormalities can act in tandem and interact in determining the severity or the susceptibility of an individual to mood disorders (Caspi et al., 2003; Zill et al., 2004; Zhou et al., 2005; Chi et al., 2013).

In this study, we utilized a mouse model of TPH2 deficiency to determine how congenital serotonergic deficiency affects the responses to chronic stress. This mouse model has single-nucleotide polymorphism (SNP) mutation of the TPH2 gene, which is analogous to that of human patients with major depressive disorder. These TPH2 knock-in (KI) mice exhibit reduced serotonin synthesis by 80%, and increased depressive- or anxiety-like behaviors (Zhang et al., 2005; Beaulieu et al., 2008; Jacobsen et al., 2012b). As HPA axis response to chronic stress can differ at different developmental time points, we also seek to characterize the differences in the adult behaviors and underlying biochemical mechanisms that occur when chronic stress paradigms are applied at different time points. The first time-point is during early life using the maternal separation (MS) paradigm (Plotsky and Meaney, 1993; Newport et al., 2002), and the second is during adulthood using the chronic mild stress paradigm (Willner, 1997; Pothion et al., 2004).

In order to elucidate the biochemical and neurochemical effects of stress on the serotonergic system, we used a pharmacological approach to directly activate the glucocorticoid receptors. Dexamethasone (Dex) is a synthetic glucocorticoid that binds to and activates the glucocorticoid receptor, with minimal mineralocorticoid receptor activation (Meaney and Aitken, 1985). Here, we used a neuronal cell line, SH-SY5Y cells, to investigate the effects of Dex in an *in vitro* system. Thereafter, we administered Dex to WT mice during two time points that were comparable to the chronic stress paradigms, one during early life from postnatal days 7–11 and another during adulthood.

The pathophysiology of depression involves complex interactions between environmental, genetic and biochemical factors. By using both WT and serotonin deficient mice to analyze the impacts of different types of chronic stress on behavior and on the serotonergic system, we hope to gain a clearer understanding of how the stress response and serotonergic systems interact to lead to depression. This in turn will allow us to identify targets for therapeutic interventions, in order to develop better therapies to combat depression.

## Materials and Methods

### Animals

C57BL/6 WT and TPH2 KI male mice were generated by heterozygous breeding and genotyped as previously described (Zhang et al., 2004). Mice were housed in a specific pathogen free environment, on a 12-h light/dark cycle (lights on 7 a.m. to 7 p.m.) and with food and water available *ad libitum*. All experiments were conducted in accordance with national guidelines for the care and use of laboratory animals for scientific purposes and with approval from the Institutional Animal Care and Use Committee (IACUC) of Duke-NUS Graduate Medical School, Singapore.

### Experimental Design for Chronic Stress During Adulthood

At 8 weeks old, mice were selected to undergo a series of mild stressors over 4 weeks (Table 1). The mild stressors that were applied included: cage tilt of approximately 45°, overnight wet bedding or bedding removal, 30 min restraint stress, 6 h water or food deprivation, cage swap and constant agitation, and isolation where mice were housed individually overnight. Reversed light cycle was introduced on Saturdays and Sundays. Tail suspension test (TST), which was performed on day 26 of the chronic mild stress paradigm, was used as a stressor and an assay for depressive-like behavior. Mice were sacrificed at 12 weeks old, blood was collected, and brain tissue samples were rapidly dissected and snap frozen. Corticosterone assay and neurochemical analyses were then carried out on the collected samples.

**Table 1:** **The experimental design of chronic mild stress in adult mice**.

	Week 1	Week 2	Week 3	Week 4
Mon	Cage tilt (overnight)	Bedding removal (overnight)	Restraint stress (30 min)	Wet bedding (overnight)
Tue	Wet bedding (overnight)	Isolation (overnight)	Cage tilt (overnight)	Water removal (6 h)
Wed	Restraint stress (30 min)	Food removal (6 h)	Food removal (6 h)	Cage tilt (overnight)
Thu	Water removal (6 h)	Cage tilt (overnight)	Wet bedding (overnight)	Cage swap and shaking
Fri	Cage swap and shaking	Cage swap and shaking	Bedding removal (overnight)	TST
Sat	Light cycle reversed	Light cycle reversed	Light cycle reversed	Light cycle reversed
Sun	Light cycle reversed	Light cycle reversed	Light cycle reversed	Light cycle reversed

### Experimental Design for Chronic Stress During Early Life (Maternal Separation)

Litters were randomly assigned into two groups, non-maternally separated (non-MS) controls and maternally separated (MS) groups. Newborn pups were subjected to MS stress for 3 h each day starting from postnatal day 1 to day 14, and this took place between 9:00 h and 15:00 h. Pups assigned to the MS group were separated from the dams and placed on a heated blanket maintained at 37°C, in plastic containers with breathable lids. After the 3 h of separation, the pups were then returned to their homecage. Control groups were left undisturbed in their home cage throughout the experiment. At the end of the procedure, all pups remained undisturbed until weaning at 3 weeks. Behavioral testing commenced when the mice reached 8 weeks of age. At the end of the behavioral experiments, control and maternally separated WT and TPH2 KI mice were sacrificed. The blood was collected, and the frontal cortex, striatum and brainstem were rapidly dissected and snap frozen. Additionally, a separate batch of non-MS and MS mice were sacrificed at 4 weeks, and brain samples were collected. Neurochemical, and gene and protein expression analyses were then carried out on brain samples.

### Experimental Design for Dexamethasone Treatment of SH-SY5Y Cell Line

The human neuroblastoma, SH-SY5Y, cell line was a gift from Dr. Lim Kah Leong (National Neuroscience Institute, Singapore). SH-SY5Y cells were cultured for 16 h in DMEM medium-high glucose (Sigma-Aldrich, St. Louis, MO, USA), pH 7.3, supplemented with 10% charcoal/dextran treated FBS (Hyclone™, USA) to remove endogenous glucocorticoids. After which, cells were treated with 100 nM Dex (Sigma-Aldrich, St. Louis, MO, USA) dissolved in dimethyl sulfoxide (DMSO) or a vehicle of DMSO for another 24 h. After treatment, the cells were pelleted and stored in −80°C until gene and protein expression analyses were carried out.

### Experimental Design for Dexamethasone Treatment of WT Mice

Dexamethasone or vehicle was administered to WT 8 week old and 7 day old mice. In 8 week old mice, Dex was administered for five consecutive days, and mice were sacrificed on the following day. In 7 day old pups, Dex was administered for five consecutive days from PND7 to PND11, and mice were sacrificed at 4 weeks of age. Dexamethasone (Sigma-Aldrich, St. Louis, MO, USA) was first dissolved in ethanol, then subsequently diluted with autoclaved water to a final concentration of 6% ethanol. 1 mg/kg of Dex was injected intraperitoneally with a 5 ml/kg injection volume. Vehicle was 6% ethanol. Corticosterone assay, neurochemical, and gene and protein expression analyses were then carried out on collected samples.

### Behavioral Testing

For all procedures, mice were brought into the testing room several hours before the test to allow for acclimatization. The behavior tests were conducted on consecutive days and in the same sequence: light/dark box, elevated zero maze, and then tail suspension. All behavioral apparatus were cleaned between each animal with surface disinfectant and 70% ethanol. In the light/dark box test, a behavioral test for anxiety (Bourin and Hascoët, 2003), mice were individually placed into the dark box (20.32 cm × 40.5 cm × 16 cm), and allowed 10 min of free exploration. Behavioral measures such as the percentage of time spent in the light box, horizontal activity in the light box, latency to enter the light box and the number of transitions between the two boxes were monitored using the Versamax program (AccuScan Instruments Inc., Columbus, OH, USA). In the elevated zero maze, the second test for anxiety (Shepherd et al., 1994; Rodriguiz and Wetsel, 2006), mice were individually placed into the closed arm and allowed 5 min for exploration. The number of transitions from one closed arm to the other closed arm and the percentage of time spent in the open arm were scored using JWatcher (UCLA, Los Angeles, CA, USA). Both control and maternally separated groups underwent TST to evaluate depressive-like behaviors. Mice were subjected to 6 min of inescapable stress of being suspended by their tail (Crowley et al., 2004). Immobility time was defined duration of time in which the force of the mouse’s movement did not exceed a set threshold as detected by a strain gauge in the Tail Suspension Chamber (Med Associates Inc., St. Albans, VT, USA).

### Corticosterone Assay

The blood collected was centrifuged at 9.0 rpm for 20 min at 4°C. Serum was collected and stored at −80°C until use. Corticosterone level was measured using Corticosterone EIA KIT (Enzo Life Sciences, Exeter, UK) following manufacturer’s protocol with the following modification: the primary antibody and conjugate were added serially, with 2 h incubation time with each substrate. After the reaction was terminated using the supplied stop solution, the plate was read with the optical density set at 405 nm using the TECAN Infinite M200 Microplate Reader (TECAN Group Ltd., Männedorf, SZ).

### High Liquid Performance Chromatography Analysis of Neurotransmitter Levels

Tissue samples of frontal cortex, striatum and brainstem tissue were weighed before being homogenized in 0.5 M perchloric acid. Following centrifugation and filtration through 0.1 μm filters (Ultrafree, Millipore, Temecula, CA, USA), the supernatants were loaded into a HPLC autosampler and pump (Dionex Ultimate 3000, ThermoFisher Scientific, Waltham, MA, USA). For separation, a mobile phase consisting a 17.5% methanol buffer (pH 3.0, 30 mM citrate-phosphate buffer, 2.1 mM octyl sodium sulfate, 0.1 mM EDTA, 10 mM NaCl), with a flow rate of 0.5 ml/min was used. Detection was by the Dionex Coulochem III Electrochemical detector (ThermoFisher Scientific Inc., Waltham, MA, USA). Calculation of 5-HT and 5-HIAA levels were based on values derived from 5-HT and 5-HIAA standards (Sigma, St. Louis, MO, USA) using the Chromeleon software (ThermoFisher Scientific Inc., Waltham, MA, USA), and were normalized to wet tissue weight for analysis. Serotonin turnover rate is taken as (amount of 5-HIAA)/(amount of 5-HT).

### Semi-Quantitative and Real-Time PCR

ProtoScript® M-MuLV First Strand cDNA Synthesis Kit (New England Biolabs, MA, USA) was used to obtain cDNA from RNA that was extracted from brain tissues and SH-SY5Y cells. Semi-quantitative PCR (qPCR) was performed on the cDNA from brain tissues using the following primers: monoamine oxidase A (MAO A) Forward (5′- ACG GAT CTG GAG AAG CCC AGT ATC - 3′), MAO A Reverse (5′ - TTC ACT TTA TCC CCA AGG AGG ACC A - 3′), mTPH2 580F (5′ - GAT GTG GCC ATG GGC TAT AAA - 3′), and mTPH2 869R (5′ - GTG CAG TGG AAT ACT CTG TAG G - 3′). mRNA expression was normalized to b-actin using the following primers: b-actin forward (5′ - ACC CAC ACT GTG CCC ATC TA - 3′) and b-actin Reverse (5′ - CGG AAC CGC TCA TTG CC - 3′). For SH-SY5Y cells, the following primers were used: hMAO A Forward (5′ - TGGAGAATCAAGAGAAGGCGAGTATCG - 3′), hMAO A Reverse (5′ - TTCACTTGGTCTCCGAGGAGGTCCA - 3′), and GAPDH Forward (5′ - TGCMTCCTGCACCACCAACT - 3′) and GAPDH Reverse (5′ - TGCCTGCTTCACCACCTTC - 3′). Real-Time qPCR was performed on SH-SY5Y cells with MAO A forward primer (5′ - TGGAGAATCAAGAGAAGGCGAGTATCG - 3′) and MAO A reverse primer (5′ - ACCAACATCTACGTAATCAACATGCTCATTC - 3′), and mRNA expression was normalized to GAPDH, using the forward primer (5′ - ATGCCATCACTGCCACYCAGAAG - 3′) and the reverse primer (5′ - TGCCAGTGAGCTTCCCGTTCAG - 3′).

#### Western Blots

Brain tissues and SH-SY5Y cell pellets were homogenized on ice in a 4% sodium dodecyl sulphate (SDS). Fifty micrograms of protein extracts were resolved on 7.5% SDS-polyacrylamide gels and transferred onto nitrocellulose membranes (Bio-Rad, CA, USA). Immunoblots of striatum and brainstem samples, and SH-SY5Y cells were probed with MAO A antibody (Santa Cruz, Dallas, TX, USA; 1:200) and Actin (Millipore, Temecula, CA, USA; 1:100000). Protein extracts from the MAO A knockout mice, which were kindly gifted by Dr. Jean Shih JC (USC, USA), were run as negative controls for MAO A antibody staining. Immunoblots of brainstem samples were further probed with glucocorticoid antibody (ThermoScientific, IL, USA; 2.5 μg/mL). Image J software (NIH, USA) was used for densitometric analyses and relative levels of proteins were normalized against actin as a loading control.

#### Statistical Analysis

Data were analyzed using the R statistical program. Data are expressed as mean ± SEM and *p* < 0.05 was considered as statistically significant. One-way or two-way ANOVAs (factors: genotype and treatment) were used to assess the behavioral, corticosterone and neurochemical data for chronic stress and MS. A two-tailed, Student’s *t*-test was used to assess all other datasets. Bonferroni-corrected pairwise comparisons were used as *post hoc* tests.

## Results

### Effects of Chronic Mild Stress Applied During Adulthood

**Figure 1:**
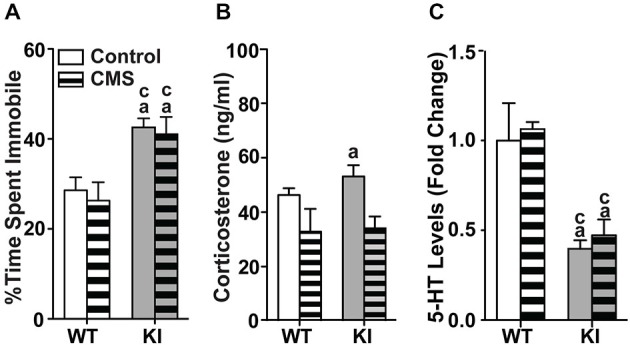
**Effects of chronic mild stress applied during adulthood. (A)** Chronic mild stress during adulthood had no effect on the time spent immobile in the tail suspension test in both WT and TPH2 KI mice. **(B)** Chronic mild stress decreased levels of serum corticosterone for both genotypes. **(C)** Serotonin levels in the brainstems of control and CMS TPH2 KI mice were lower than control WT mice. No differences in serotonin levels were detected between control and CMS WT mice. *N* = 10–20 mice/genotype/treatment condition.^ a^*p* < 0.05 from WT control, ^c^*p* < 0.05 from WT stress. Figure key: White, open bar—WT non-stressed controls; white horizontal striped bar—WT CMS; gray open bar—TPH2 KI, non-stressed controls; gray, horizontal striped bar—TPH2 KI CMS.

Exposure to 4 weeks of chronic unpredictable stress (CMS) during adulthood did not affect the behaviors of adult WT and TPH2 KI mice, as a two-way ANOVA for the percentage of time spent immobile in the TST only showed a significant main effect of genotype (*F*_(1,24)_ = 18.4, *p* < 0.001; Figure 1A). Both CMS and control TPH2 KI mice exhibited increased depressive-like behaviors than their WT counterparts, and these two TPH2 KI groups did not behave differently from each other.

At baseline, corticosterone levels in TPH2 KI mice were significantly higher than WT controls (*F*_(1,27)_ = 7.432, *p* = 0.0111; Figure 1B). The mean corticosterone concentration in the serum of WT mice was 41908 (SE = 2546) pg/ml and of TPH2 KI mice was 56505 (SE = 4590) pg/ml. In CMS groups, there was a significant main effect of CMS on serum corticosterone levels (*F*_(1,46)_ = 9.21, *p* = 0.00396), with no significant main effect of genotype or interaction. Serotonin levels in brainstem tissue showed significant main effects of genotype (*F*_(1,78)_ = 32.3, *p* < 0.0001; Figure 1C), with no significant main effect of CMS or interaction.

### Effects of Maternal Separation on Adult Behaviors

Newborn pups were subjected to MS stress for 3 h each day starting from postnatal day 1 to day 14, after which, the pups were left undisturbed until adulthood at 2 months old. At adulthood, the MS pups and their controls (non-MS) were subjected to a series of behavior tests.

**Figure 2:**
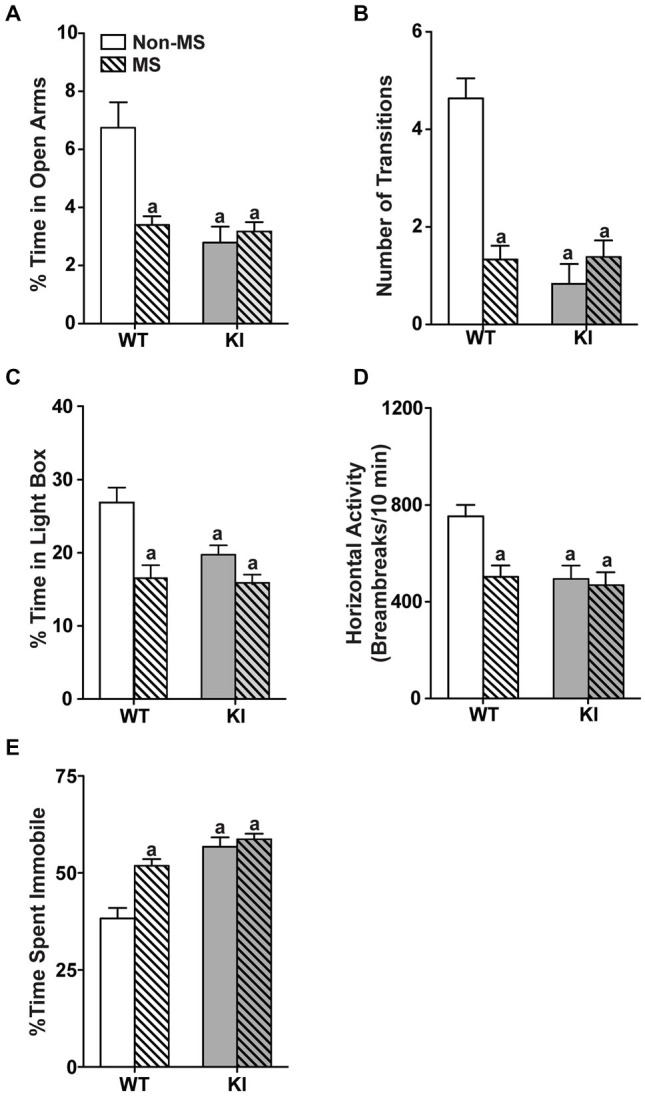
**Effects of maternal separation on adult behaviors**. In panels **(A–E)**, non-MS and MS TPH2 KI exhibit increased anxiety-like and depressive-like behaviors compared to non-MS WT in all behavioral tests. In the zero maze, MS WT had reduced percentage of time spent in the open arm **(A)** and number of transitions between the two closed arms **(B)** compared to non-MS WT. In the light/dark box, MS WT had reduced percentage of time spent in the light box **(C)** and horizontal activity in the light box **(D)** compared to non-MS WT. **(E)** MS increased the percentage of time spent immobile in the tail suspension test in WT, but not in TPH2 KI mice. *N* = 10–30 mice/genotype/treatment condition. ^a^*p* < 0.05 from WT control. Figure key: White, open bar—WT non-stressed controls; white, diagonal striped bar—WT MS; gray open bar—TPH2 KI, non-stressed controls; gray, diagonal striped bar—TPH2 KI MS.

In the zero maze (Figures 2A,B), there were significant main effects of genotype (time: *F*_(1,70)_ = 19.2, *p* < 0.0001; transition: *F*_(1,70)_ = 23.9, *p* < 0.0001) and MS (time: *F*_(1,70)_ = 9.57, *p* = 0.00279; transition: *F*_(1,70)_ = 12.9, *p* = 0.0006), with significant genotype by MS interaction (time: *F*_(1,70)_ = 16.0, *p* = 0.000149; transition: *F*_(1,70)_ = 25.4, *p* < 0.0001). Non-MS TPH2 KI mice showed decreased time spent in the open arms (Figure 2A) and reduced number of transitions between the open arms (Figure 2B) compared to non-MS WT mice. MS WT mice also had significantly decreased exploratory measures in the zero maze compared to non-MS WT mice (*p*s < 0.001) and exhibited similar levels of anxiety-like behaviors to non-MS and MS TPH2 KI mice, which in turn were not significantly different from each other. In the light/dark box (Figures 2C,D), the two-way ANOVA showed a significant main effects of genotype (time: *F*_(1,75)_ = 6.04, *p* = 0.00163; horizontal activity: *F*_(1,75)_ = 5.71, *p* = 0.0194) and MS (time: *F*_(1,75)_ = 20.5, *p* < 0.0001; horizontal activity: *F*_(1,75)_ = 5.21, *p* = 0.0253). The genotype by MS interaction term was significant for both percentage time spent (*F*_(1,75)_ = 4.11, *p* = 0.0444) and horizontal activity (*F*_(1,75)_ = 5.10, *p* = 0.0268). Pairwise comparisons showed that MS WT, non-MS TPH2 KI and MS TPH2 KI had significantly reduced percent time spent (Figure 2C) and horizontal activity (Figure 2D) in the light box when compared to non-MS WT (*p*s < 0.01). MS WT mice had similar exploratory levels in the light box as non-MS and MS TPH2 KI mice.

In the TST (Figure 2E), there were significant main effects of genotype (*F*_(1,104)_ = 39.3, *p* < 0.001) and MS treatment (*F*_(1,104)_ = 14.8, *p* < 0.001), with significant genotype by MS treatment interaction (*F*_(1,104)_ = 8.23, *p* = 0.005). Within the non-MS group, TPH2 KI mice remained immobile longer than WT mice (*p* < 0.0001). WT and TPH2 KI mice that had undergone MS showed increased depressive-like behaviors compared to non-MS WT mice (*p*s < 0.0001), and did not significantly differ from non-MS TPH2 KI mice.

### Effects of Maternal Separation on Serum Corticosterone and the Serotonergic System in Adulthood

**Figure 3:**
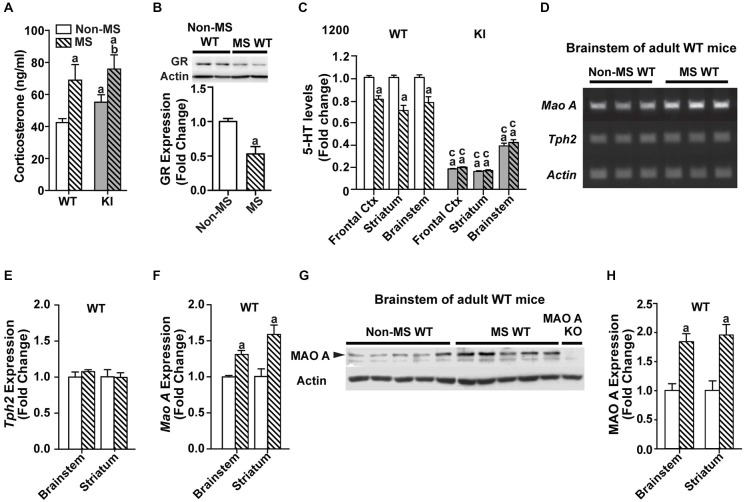
**Effects of maternal separation on serum corticosterone and the serotonergic system in adulthood. (A)** Corticosterone levels of both MS WT and TPH2 KI mice were higher than their respective non-MS controls. **(B)** Expression of glucocorticoid receptors in adult WT mice was reduced after undergoing maternal separation. **(C)** Serotonin levels in three brain areas of adult mice, the frontal cortex, striatum and brainstem, were lowered in MS WT than non-MS WT. Both non-MS and MS TPH2 KI had significantly lowered serotonin levels in all three brain areas compared to control or MS WT mice. **(D)** Representative results of the semi-PCR for TPH2 and MAO A in adult non-MS and MS WT mice. TPH2 expression remained unchanged **(E)**, whereas MAO A expression was increased **(F)**, in both the brainstem and striatum of MS compared to non-MS WT adult mice. **(G)** Representative blot of MAO A protein expression in the brainstem of WT adult mice. **(H)** Western blot analyses of adult brainstem and striatum showed that MAO A protein expression was increased in MS WT mice relative to non-MS controls. For serum corticosterone levels, *N* = 10–20 mice/genotype/treatment condition. For panels **(B)** to **(H)**, *N* = 5 mice/genotype/treatment condition. ^a^*p* < 0.05 from WT control, ^b^*p* < 0.05 from KI control, ^c^*p* < 0.05 from WT stress. Figure key: White, open bar—WT non-stressed controls; white, diagonal striped bar—WT MS; gray open bar—TPH2 KI, non-stressed controls; gray, diagonal striped bar—TPH2 KI MS.

In the MS groups, corticosterone measurements showed significant effects of MS treatment (*F*_(1,58)_ = 18.1, *p* < 0.001), with no significant main effect of genotype or interaction (Figure 3A). Within genotype, MS WT mice had higher levels of corticosterone than non-MS WT mice (*p* = 0.00761), whereas there was no significant difference in corticosterone levels between non-MS and MS TPH2 KI mice. Additionally, MS WT and MS TPH2 KI mice had similar levels of corticosterone. In MS WT mice, GR expression levels were reduced at adulthood, compared to controls (Figure 3B; *p* = 0.006).

Neurochemical analyses of tissue levels of serotonin in the frontal cortex, striatum and brainstem (Figure 3C) of non-MS and MS, WT and TPH2 KI mice showed that there was a significant main effect of genotype (Frontal cortex: *F*_(1,32)_ = 1150, *p* < 0.001; Striatum: *F*_(1,36)_ = 223, *p* < 0.001; Brainstem: *F*_(1,29)_ = 220 *p* < 0.001). The other main effect of MS treatment also significantly affected tissue levels of serotonin (Frontal cortex: *F*_(1,32)_ = 14.5, *p* < 0.001; Striatum: *F*_(1,36)_ = 9.53, *p* = 0.00388; Brainstem: *F*_(1,29)_ = 13.9, *p* < 0.001), and there was a significant genotype by treatment interaction in all three brain regions (Frontal cortex: *F*_(1,32)_ = 23.1, *p* < 0.0001; Striatum: *F*_(1,36)_ = 10.1, *p* = 0.00308; Brainstem: *F*_(1,29)_ = 8.65, *p* = 0.00638). Pair-wise comparisons showed that non-MS TPH2 KI mice showed significantly lower levels of serotonin in the tissue compared to WT mice in all three brain regions (*p*s < 0.001). Within genotype, MS WT mice showed significant reduction in serotonin levels in all three brain regions compared to non-MS WT counterparts (*p*s < 0.001), whereas there were no significant difference in serotonin levels between the non-MS and MS TPH2 KI mice. Even though serotonin levels of maternally separated WT mice were reduced, the levels in the frontal cortex, striatum and brainstem were still higher than both non-MS and MS TPH2 KI mice (*p*s < 0.001).

### Maternally Separated WT Mice Had Increased MAO A Activity During Adulthood

To determine the underlying cause of the decreased serotonin levels observed in MS WT mice, the expression levels of the rate-limiting enzyme in serotonin production, TPH2, and the enzyme that breaks down serotonin, MAO A, were measured. The semi-qPCR results showed that TPH2 mRNA expression levels in the brainstem and striatum (Figures 3D,E) of MS WT mice were not different from non-MS WT mice, whereas mRNA expression of MAO A is increased in MS WT mice (Figures 3D,F; *p*s < 0.007). The results of the semi-qPCR were corroborated by western blot analyses of MAO A expression in adult WT mice, where an increase in MAO A expression in maternally separated WT mice, relative to controls, was observed in both the brainstem and striatum (Figures 3G,H; *p*s < 0.002).

### Maternally Separated WT Mice Had Increased MAO A Activity at 4 Weeks of Age

**Figure 4:**
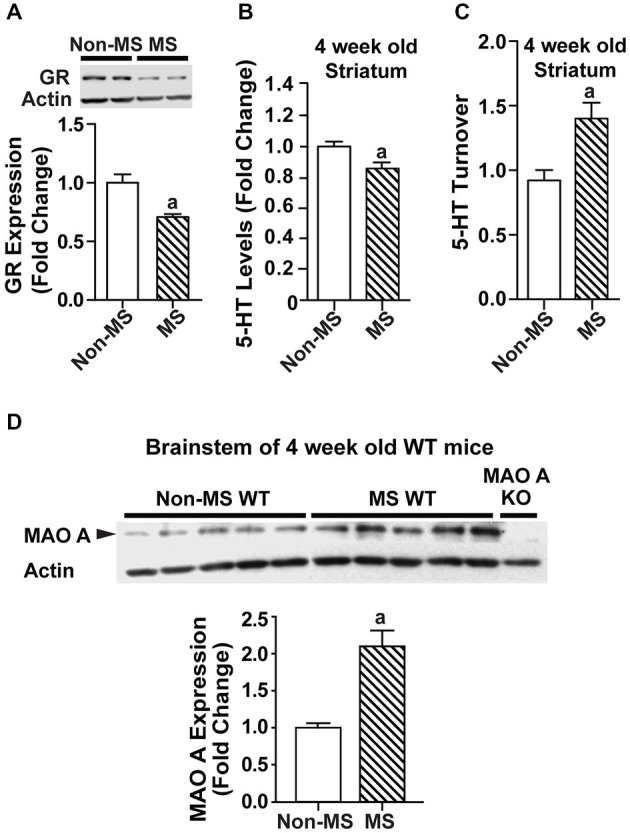
**Effects of maternal separation on glucocorticoid receptor expression and the serotonergic system in 4-week-old mice. (A)** Expression of glucocorticoid receptors in 4-week-old WT mice was reduced in those that underwent maternal separation. **(B)** Serotonin level in the striatum of 4-week-old MS WT mice was lower than that of non-MS WT mice, and **(C)** serotonin turnover was increased. **(D)** Representative blot of MAO A protein expression in the brainstem of 4-week-old WT mice, and quantification of western blot analyses of 4-week-old brainstem, which showed that MAO A protein expression was increased in MS WT mice compared to controls. *N* = 5 mice/genotype/treatment condition. ^a^*p* < 0.05 from WT control. Figure key: White, open bar—WT, non-MS controls; diagonal striped bar—WT, MS.

In order to determine the point at which the biochemical changes occurred, a separate batch of MS WT mice and their controls were sacrificed at a younger timepoint of 4 weeks, and their brain tissues harvested for biochemical analyses. Relative to control WT mice, glucocorticoid receptor levels were reduced in MS WT at 4 weeks of age (Figure 4A; *p* < 0.05). In addition, serotonin levels were found to be lowered in the striatum of 4-week-old MS mice compared to controls (*F*_(1,25)_ = 9.44, *p* = 0.00508; Figure 4B), with increased serotonin turnover (*F*_(1,10)_ = 13.5, *p* = 0.00115; Figure 4C). The increased MAO A expression was also found in the brainstem of 4-week-old MS WT mice (Figure 4D; *p* = 0.0054). Thus, the reduction of serotonin levels in the brain following MS is likely to be due to the higher expression of MAO A, which resulted in increased serotonin turnover. In addition, this perturbed expression level of MAO A is present in 4-week-old MS WT mice and persists into adulthood.

### Dexamethasone Treatment Increased MAO A Expression in SH-SY5Y Cells

**Figure 5:**
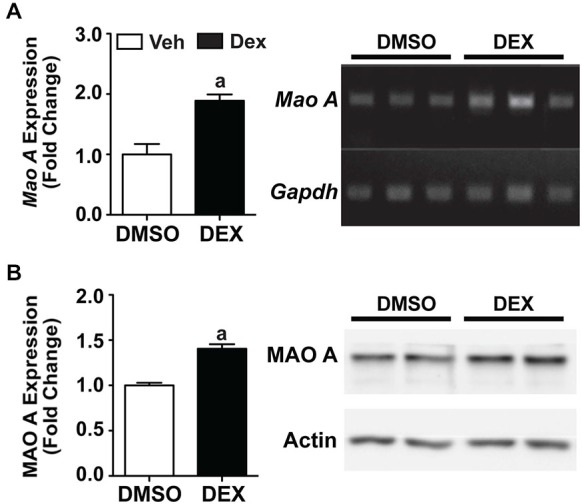
**Twenty four hours Dex treatment increased MAO A expression in SH-SY5Y cells**. Quantification of mRNA expression **(A)** and protein expression **(B)** showed that MAO A increased after 24 h treatment with Dex (black bars) compared to DMSO treatment (white bars). Representative results from semi-PCR and western blots are shown in panels **(A)** and **(B)** respectively. *N* = 3 replicates. ^a^*p* < 0.05 from DMSO control. Figure key: White bar—DMSO-treated; black bar—Dex-treated.

We then used an *in vitro* system to investigate whether a pharmacological stressor, Dex, will result in similar biochemical responses that was observed in maternally separated WT mice. Using neuronal SH-SY5Y cells, we showed that 24 h Dex treatment resulted in significant increases in both mRNA and protein expression levels of MAO A compared to the DMSO-treated controls (Figure 5; *p*s < 0.01).

### Dexamethasone Treatment During Adulthood did not Affect the Serotonergic System Serotonin

**Figure 6:**
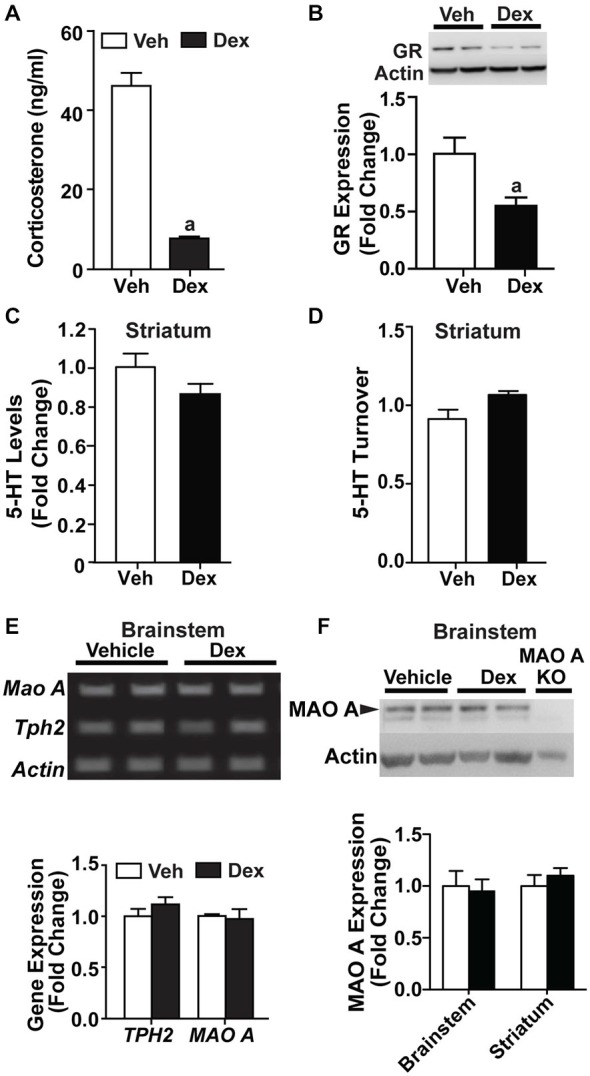
**Dex treatment administered during adulthood did not affect the serotonergic system. (A)** 1 mg/kg Dex treatment for 5 days resulted in a suppression of corticosterone in adults. **(B)** Glucocorticoid receptor expression was reduced in Dex-treated adult mice. **(C,D)** There was no detectable difference in serotonin level and turnover rate in the striatum of adult, Dex-treated WT mice compared to controls. **(E)** Representative results and quantification from semi-PCR analyses showing TPH2 and MAO A expression in adult mice. No change was detected in TPH2 and MAO A gene expression in the brainstem of adult, Dex-treated WT mice compared to controls. **(F)** A representative western blot showing MAO A expression in the brainstem from adult WT mice, and quantification of the western blot results showing no change in MAO A protein expression in the brainstem and striatum of adult, Dex-treated WT mice compared to controls. *N* = 12–15 mice/treatment condition for corticosterone and HPLC analyses; *N* = 5 mice/treatment condition for western blots and semi-PCR. ^a^*p* < 0.05 from Veh control. Figure key: White bar—Veh treated; black bar—Dex treated.

Following the increased MAO A expression observed in SH-SY5Y cells, we treated adult WT mice for 5 days with 1 mg/kg Dex. Corticosterone levels (*p* < 0.0001; Figure 6A) and glucocorticoid receptor expression (*p* < 0.05; Figure 6B) were significantly reduced in Dex-treated WT mice compared to vehicle-treated controls.

Neurochemical analyses showed no detectable change in serotonin levels and turnover rate in the striatum of Dex-treated WT mice relative to controls (Figures 6C,D). No change was detected in the mRNA expression of TPH2 and MAO A (Figure 6E). Additionally, the protein expression level of MAO A remained unchanged in the brainstem and striatum (Figure 6F). Hence, Dex treatment, when applied in adult mice as a pharmacological manipulation to activate glucocorticoid receptors, did not result in a dysregulation in serotonin levels and MAO A expression levels when administered during adulthood.

### Dexamethasone Treatment During Postnatal Days 7–11 Caused Dysregulations in the Serotonergic System at 4 Weeks of Age

**Figure 7:**
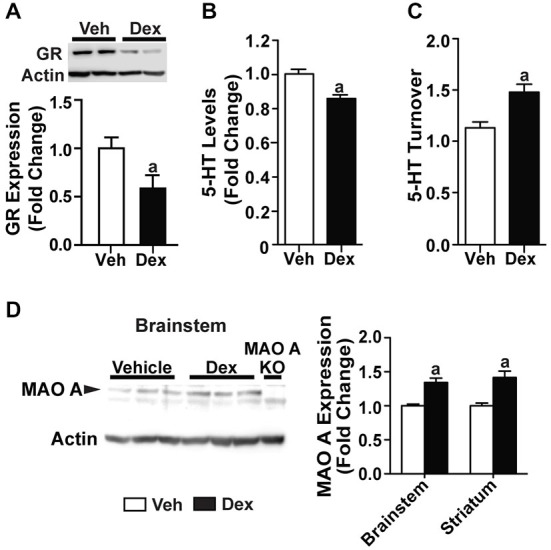
**Dex treatment administered during postnatal days 7–11 caused dysregulations in the serotonergic system at 4 weeks of age. (A)** Expression of glucocorticoid receptors in 4-week-old mice was reduced in those that were treated with Dex during PND7–11. **(B,C)** At 4 weeks of age, serotonin level was reduced and turnover rate was increased in mice that were treated with Dex during PND7–11 compared to controls. **(D)** Representative western blot showing MAO A expression in the brainstem of 4-week-old mice, and quantification of western blot results from the brainstem and striatum of 4-week-old mice, which showed an increased in MAO A protein expression in mice that were treated with Dex during PND7–11. *N* = 5 mice/treatment condition. ^a^*p* < 0.05 from Veh control. Figure key: White bar—Veh treated/Non-MS; black bar—Dex treated.

Previous studies have shown that postnatal Dex treatment, which began at PND7, resulted in a decrease of serotonin levels (Slotkin et al., 2006). Here, we investigated if Dex treatment during this postnatal period would affect MAO A expression, and hence serotonin levels and turnover rate. Mice are sacrificed at 4 weeks of age to show that biochemical changes were already present even before adulthood. Dex treatment during postnatal days 7–11 significantly reduced glucocorticoid receptor expression in 4-week-old WT mice (*p* < 0.05; Figure 7A). Neurochemical analyses showed that serotonin levels in the striatum of PND7–11 Dex-treated mice were lower (Figure 7B; *p* = 0.000523), whereas serotonin turnover was higher, than that of controls (Figure 7C; *p* = 0.00115). In addition, PND7–11 Dex-treated WT mice had significantly higher MAO A protein expression levels in the brainstem and striatum compared to DMSO-treated WT mice (Figure 7D; *p* < 0.005).

## Discussion

The key findings of this study are firstly that environmental stressors, when applied during the early postnatal period, will induce anxiety- and depressive-like behaviors through dysregulations of the serotonergic system in WT mice, with no detectable effects in TPH2 KI mice. Secondly, both environmental and pharmacological stressors, when applied during the early postnatal period, results in dysregulations of the serotonergic system of WT mice. The reduced serotonin level in the brains of WT mice that have been treated during early life with either environmental or pharmacological stressors can be attributed to an increase in MAO A expression.

### Chronic Mild Stress and Maternal Separation Had Different Effects on the Behavior and Serotonergic System of WT Mice

Chronic mild stress has often been used as a model of depression as it is able to induce anhedonia, as characterized by the lack of sucrose preference, which is a hallmark symptom of depression (Willner, 1997, 2005; Pothion et al., 2004). Here, we found that chronic mild stress in adult WT mice resulted in a suppression of corticosterone levels, which may be due to adaptation following repeated stress episodes, as has been shown previously (Chen and Herbert, 1995; Chung et al., 1999). However, we did not observe any increase in behavioral despair, as assessed by the TST. In addition, there were no detectable differences in levels of serotonin in the brains of controls vs. stressed groups. Our findings suggest that chronic mild stress may not be a suitable stressor to reliably elicit a depressive-like behavioral response in the WT mice, or that the C57/BL6J genetic background of our mice has masked the effects of the chronic mild stress manipulation (Willner, 2005; Mineur et al., 2006).

In maternally separated WT mice, we showed that the manipulation resulted in the exhibition of anxiety- and depressive-like behaviors. The MS paradigm is often used to mimic neglect (Meaney et al., 1985; Plotsky and Meaney, 1993; Newport et al., 2002), and there is a wealth of studies showing that MS in WT mice and rats chronically impairs emotional and neuroendocrine responses. Our results are corroborated by many previous studies, which showed that MS of mouse or rat pups caused long-term changes in behaviors associated with anxiety and depression, even if compensatory postnatal maternal care was present (Plotsky and Meaney, 1993; Wigger and Neumann, 1999; MacQueen et al., 2003; Romeo et al., 2003; Veenema et al., 2006; Wei et al., 2010; Niwa et al., 2011; Sachs et al., 2013).

Additionally, the disrupted maternal care in WT mice may cause dysregulations in the corticosterone levels, which may in turn affect the growth and development of the central nervous system, and subsequently behavior in adulthood (Plotsky and Meaney, 1993; Ladd et al., 1996; Huot et al., 2002; Nishi et al., 2013). Here, we observed an increase in serum corticosterone levels of adult WT mice that underwent MS, which is in congruence with past studies showing that early life stress, such as repeated MS and isolation rearing, results in elevated levels of circulating corticosterone (Lippmann et al., 2007; Niwa et al., 2011). We also showed the glucocorticoid receptor expression was reduced in maternally separated WT mice compared to controls, which is likely due to the presence of elevated corticosterone levels (Sapolsky et al., 1984; Meaney et al., 1985; Rosewicz et al., 1988).

Previous studies have shown that the type of stressors applied can affect serotonin levels in brain tissue (Kirby et al., 1995, 1997). Here, we found that MS resulted in lowered serotonin levels in three brain areas, the brainstem, striatum and frontal cortex in adult WT mice. Furthermore, analysis of brain samples from an earlier timepoint of 4 weeks showed that serotonin levels were already reduced, and serotonin turnover rate was increased. Hence, we showed that serotonin dysregulation was present in MS WT mice at periadolescence, and persisted into adulthood.

The effects of MS on serotonin levels and turnover suggested that serotonin synthesis or breakdown was dysregulated. As such, we characterized the expression levels of TPH2 and MAO A in 4 week old and adult WT, maternally separated and control mice. We observed an upregulation of the mRNA and protein expression levels of MAO A, in both age groups of maternally separated WT mice. Similarly, an earlier study using chronic socially defeated rats showed that chronic social defeat stress caused an elevation in corticosterone levels, decrease in serotonin levels and increased MAO A activity in the cortex and thalamus (Grunewald et al., 2012). Interestingly, TPH2 levels in maternally separated WT mice were no different from that of controls, even though previous studies have shown that both acute and chronic environmental (sound) stressors or pharmacological (corticosterone hemisuccinate) stressors can affect TPH2 activity (Azmitia and McEwen, 1976; Boadle-Biber et al., 1989). However, the results from a study on rats that were exposed to stress in early life were in line with what we have found here, essentially that corticosterone level was increased, serotonin level was reduced and serotonin turnover was increased, with no changes in the expression levels of TPH2 (Mesquita et al., 2007). Hence, we suggest that the type of stressor used and the timepoint of application of the stressor play a role in determining whether TPH2 activity is affected. Here, we find that MS only affects MAO A, and not TPH2 activity. Therefore, we hypothesized that the elevation of corticosterone levels in maternally separated mice may be the cause of the observed upregulation of MAO A, which in turn increases serotonin turnover.

Our results show that MS results in the exhibition of adverse behaviors, and dysregulates corticosterone levels and the serotonergic system of adult WT mice, whereas chronic mild stress did not. Taken together, the presence of a critical window during the developmental period may be another plausible explanation for the lack of perturbed behavioral and neurochemical regulation in WT mice that underwent chronic mild stress. Perhaps when chronic environmental stressors are applied during early life (first 14 days), they will cause long-lasting alterations in serum corticosterone level and the serotonergic system, which in turn result in the manifestations of aberrant behaviors and decreased serotonin levels in the brain. This idea of a critical period for stressors to have permanent effects on the serotonergic system has been shown previously (Slotkin et al., 2006).

### Chronic Mild Stress and Maternal Separation did not Affect the Behaviors and Serotonergic System of TPH2 KI Mice

For TPH2 KI mice, we found that they had slightly higher levels of basal corticosterone than their WT counterparts. Although this was not previously observed (Sachs et al., 2013), our results are in congruence with previous studies using pharmacological manipulations, which have shown that prolonged serotonin depletion in rodents resulted in elevated corticosterone levels (Baumann et al., 1998; Chung et al., 1999; Franklin et al., 2011). We found that corticosterone levels were suppressed in TPH2 KI mice that were exposed to chronic mild stress and elevated in maternally separated TPH2 KI mice relative to non-stressed TPH2 KI mice. Thus, the corticosterone response of TPH2 KI mice to stress follows a pattern similar to that of WT mice that underwent either chronic mild stress or MS.

The unexpected findings of our study were that MS did not give rise to behavioral changes or serotonergic perturbations in TPH2 KI mice. The lack of observable behavioral changes in TPH2 KI mice after MS has also been observed in a previous study (Sachs et al., 2013). We suggest that this may be because the TPH2 KI mice, due to their reduced serotonin levels, already show anxiety- and depressive-like behaviors, and reduced serotonin levels at baseline (Zhang et al., 2005; Beaulieu et al., 2008; Jacobsen et al., 2012b), such that it is physiologically not possible to show exacerbated anxiety- or depressive-like phenotype in response to MS. However, MS has been shown to affect other aspects of behavior in TPH2 KI mice, such as impaired behavioral inhibition (Sachs et al., 2013).

### Effects of Dexamethasone as a Pharmacological Stressor on the Serotonergic System

In order to test whether the increased MAO A expression in maternally separated WT mice is directly due to the activation of glucocorticoid receptors as a result of the elevated corticosterone levels, we used Dex, a synthetic glucocorticoid, to induce a stress response in an *in vitro* system as a start. Using SH-SY5Y neuronal cell line, our results, which were in line with that of previous studies, showed an upregulation of MAO A mRNA and protein expression following Dex treatment, likely through the glucocorticoid-mediated activation of the MAO A promoter (Manoli et al., 2005; Ou et al., 2006; Grunewald et al., 2012).

In order to test our hypothesis that there is a critical developmental period during which chronic environmental stressors caused the observed perturbations in the serotonergic system, we treated PND7 or adult WT mice with 5 days of Dex. These timepoints were chosen to mirror the timepoints for MS and chronic mild stress. In both groups of Dex-treated mice, we observed similar reductions in glucocorticoid receptor expression. Interestingly, WT mice that were treated with Dex during PND7–11, but not those treated during adulthood, showed an increased in MAO A gene and protein expression, with the corresponding reduced serotonin levels and increased serotonin turnover. This further supports the idea that stressors when applied during a critical developmental period, will result in long-lasting, adverse effects on serotonergic regulation, thereby leading to undesirable behavioral outcomes.

### Concluding Remarks

Taken together, our results showed that stressors, be it environmental or pharmacological, would have adverse effects when applied during the critical postnatal period of at least from day 1–14, or perhaps the narrower day 7–11, but not during adulthood. To our knowledge, this is the first study showing that the interplays between early life stress and serotonergic homeostasis occur through the glucocorticoid regulation of MAO A, and not TPH2, expression. This leads to long-lasting perturbations in the serotonergic system, and results in anxiety- and depressive-like behaviors.

Our results have clinical implications, because an elevation of MAO A has been found in patients with major depressive disorder (Meyer et al., 2009; Chiuccariello et al., 2014) and is a contributing factor to postpartum depression (Sacher et al., 2010). This suggests that maternally separated or postnatal Dex-treated WT mice can be used as a valid disease model to study the underlying biological dysregulations of this group of depressive disorders. It also stands to reason that the adverse effects of early life chronic stress that we have observed can be rescued by the direct manipulation of MAO A activity. Indeed, there has been an increasing focus on the use of second generation, reversible MAO A inhibitors for the treatment of drug-resistant depression patients (Lum and Stahl, 2012; Schwartz, 2013; Shulman et al., 2013). As such, it would be interesting for future studies to investigate whether the long-lasting of effects of MS can be antagonized by the application of MAO A inhibitors, or whether lacking a functional MAO A gene can confer resistance to stressors (Popova et al., 2006). In addition, it would be interesting to follow-up on whether treating WT mice with a glucocorticoid receptor antagonist during the MS manipulation will prevent the development of the anxiety-like and depressive-like behaviors, and perturbations of the serotonergic system. Additionally, it has been found that MS affects NMDA receptor 1 expression (Sachs et al., 2013), and that there is evidence of interplay between MAO A and NMDA receptors (Kosenko and Kaminsky, 2009; Bortolato et al., 2012). Given these evidence, using this mouse model of early life environmental or pharmacological stress may also be useful in elucidating and understanding the effects of NMDA receptor antagonists, such as ketamine, which have recently demonstrated promise in the treatment of drug-resistant depression (Zarate et al., 2006; Autry et al., 2011).

## Conflict of Interest Statement

The authors declare that the research was conducted in the absence of any commercial or financial relationships that could be construed as a potential conflict of interest.
